# The protective role of sphingosine-1-phosphate against the action of the vascular disrupting agent combretastatin A-4 3-*O*-phosphate

**DOI:** 10.18632/oncotarget.21172

**Published:** 2017-09-21

**Authors:** Joanna Shepherd, Matthew Fisher, Abigail Welford, Donald M. McDonald, Chryso Kanthou, Gillian M. Tozer

**Affiliations:** ^1^ Current/Present address: School of Clinical Dentistry, The University of Sheffield, Claremont Crescent, Sheffield, UK; ^2^ Tumour Microcirculation Group, The University of Sheffield, Department of Oncology and Metabolism, School of Medicine, Sheffield, UK; ^3^ UCSF Comprehensive Cancer Center, Cardiovascular Research Institute, and Department of Anatomy, University of California, San Francisco, CA, USA

**Keywords:** sphingosine-1-phosphate, combretastatin, VE-cadherin, tumour microcirculation, adherens junctions

## Abstract

Solid tumours vary in sensitivity to the vascular disrupting agent combretastatin A-4 3-*O*-phosphate (CA4P), but underlying factors are poorly understood. The signaling sphingolipid, sphingosine-1-phosphate (S1P), promotes vascular barrier integrity by promoting assembly of VE-cadherin/β-catenin complexes. We tested the hypothesis that tumour pre-treatment with S1P would render tumours less susceptible to CA4P.

S1P (1μM) pretreatment attenuated an increase in endothelial cell (HUVEC) monolayer permeability induced by 10μM CA4P. Intravenously administered S1P (8mg/kg/hr for 20 minutes then 2mg/kg/hr for 40 minutes), reduced CA4P-induced (30mg/kg) blood flow shut-down in fibrosarcoma tumours in SCID mice (n≥7 per group), as measured by tumour retention of an intravenously administered fluorescent lectin. A trend towards *in vivo* protection was also found using laser Doppler flowmetry. Immunohistochemical staining of tumours *ex vivo* revealed disrupted patterns of VE-cadherin in vasculature of mice treated with CA4P, which were decreased by pretreatment with S1P. S1P treatment also stabilized N-cadherin junctions between endothelial cells and smooth muscle cells in culture, and stabilized tubulin filaments in HUVEC monolayers.

We conclude that the rapid shutdown of tumour microvasculature by CA4P is due in part to disruption of adherens junctions and that S1P has a protective effect on both adherens junctions and the endothelial cell cytoskeleton.

## INTRODUCTION

Tumour blood vessels tend to be disorganized, immature and hyperpermeable, with the result that tumour microvasculature has become a key target in anti-cancer therapy. The tumour vascular disrupting agent (VDA) combretastatin A-4 3-*O*-phosphate (CA4P; fosbretabulin) is a microtubule-binding agent, which induces rapid and selective tumour vascular shutdown and secondary tumour cell death [reviewed in 1, 2]. It is currently in phase I-III clinical trials in combination therapy, most notably with bevacizumab, in a variety of tumour types (http://www.mateon.com/) [3–6]. The heightened sensitivity of tumour-associated blood vessels to CA4P is not fully understood. Although these vessels have a relatively high endothelial cell proliferation rate compared to those of normal tissues [7–9] and CA4P has anti-proliferative effects on dividing endothelial cells after prolonged exposure [10], the differences in proliferative capacity cannot account for the rapid blood flow shutdown observed in tumours following VDA administration [11]. The relatively short plasma half-life of CA4P (~22 minutes) suggests that morphological and functional effects upon the cytoskeleton rather than cytotoxicity mediate the *in vivo* effects of CA4P on tumour blood flow [11]. The main aim of the current study was to investigate how the stability of vascular endothelial cadherin (VE-cadherin)-associated endothelial cell junctions in tumors influences the rapid tumor vascular response to CA4P.

Endothelial shape and function are maintained by the cytoskeleton, a key target for tubulin-binding VDAs [12]. CA4P was found to induce actin stress fibres, focal adhesions and an increase in permeability of endothelial cell monolayers in culture, triggered by microtubule disruption and mediated by the small GTPase Rho and Rho kinase [12, 13]. Furthermore, a CA4P-induced increase in solid tumour vascular permeability has been implicated in the drug's mechanism of action *in vivo* [14]. Intercellular adherens junctions are critical in maintaining vascular integrity and it is well established that the permeability of the endothelium is regulated by the endothelial cell-specific adherens junction molecule, vascular endothelial cadherin (VE-cadherin), which directly links endothelial cells to the actin cytoskeleton [15–18]. This interface is maintained via the interactions of the cytoplasmic domain of VE-cadherin with 3 related proteins (β-catenin, p120 and plakoglobulin) anchored to the actin cytoskeleton [15]. Vincent *et al* hypothesized that CA4P disrupts the VE-cadherin/β-catenin association, required for endothelial cell adhesion and survival during neovessel assembly and re-modeling, which may lead to rapid vascular collapse and tumour necrosis [17]. This rapid disengagement of the VE-cadherin/β-catenin complex and consequential rearrangement of the cytoskeleton would provide an explanation for both the immediate induction of CA4P-mediated vascular collapse and the later cytotoxic effects of the agent. Differences in the stability of VE-cadherin junctions or its association to these proteins could explain variations in endothelium permeability, and therefore why different tumours and cell lines display varying levels of susceptibility to CA4P.

N-cadherin is an additional adherens junctional protein, which is expressed on both endothelial cells and mural cells including pericytes and vascular smooth muscle cells (SMC). In the vasculature, it is involved in intercellular adhesion interactions between endothelial cells and mural cells [19]. Since investiture of pericytes to the tumour vasculature is important in vascular stabilization and maturation, it is possible that CA4P targets N-cadherin mediated intercellular junctions in addition to endothelial cell - endothelial cell interactions in the process of vascular destabilisation.

Sphingosine-1-phosphate (S1P) is a bioactive lipid mediator released, for example, at sites of endothelial injury by cells including activated platelets, and has been implicated in numerous cancer pathways [20]. It exerts its effects by binding to the G-protein coupled receptors S1P1-5 [21], which are expressed in a tissue-specific manner. Endothelial cells display the receptor S1P1, and ligation of S1P to S1P1 at physiological concentrations (10 nM – 2 μM) elicits a variety of responses, including the enhancement of barrier function by stimulation of Rho- and Rac-dependent assembly of adherens junctions [22]. S1P induces the enhancement of VE-cadherin interactions with ɑ- and β-catenin [23] and the translocation of VE-cadherin to endothelial cell junctions [21]. In the mesenteric microvasculature *in situ*, S1P was shown to stabilize the vasculature against an acute increase in permeability by preventing the rearrangement of VE-cadherin, via Rac-dependent pathways, that was otherwise induced by platelet activating factor or bradykinin [24]. Indeed, degradation of the receptor S1P1 (by a phosphorylated derivative of FTY720, an analogue of S1P), thereby preventing S1P signaling, resulted in vascular leak in mice [25].

Here, we hypothesised that disruption of tumour adherens junctions is a primary mechanism of action for CA4P *in vivo*, triggering blood flow reduction, and that this disruption could be attenuated by pretreatment with sphingosine-1-phosphate (S1P). We used a mouse fibrosarcoma cell line, fs120, which expresses the VEGF120 isoform of vascular endothelial growth factor A (VEGF-A) in isolation [26]. Solid fs120 tumors are characterized by an immature leaky vasculature with little pericyte investiture, which is prone to haemorrhage and sensitive to CA4P treatment [26]. We demonstrate the effect of CA4P on VE-cadherin adherens junctions *in vivo* in fs120 tumours, using immunofluorescence staining for VE-cadherin on frozen tissue sections. We stabilized junctions prior to CA4P insult both *in vitro* and *in vivo* by pre-treatment with S1P. To establish the effects of combined S1P and CA4P treatment on tumour perfusion *in vivo*, we monitored changes in red cell flux within fs120 tumours of live animals following treatment and measured a ‘tumour perfusion index’ in excised tumours *ex vivo*. We also demonstrate effects of CA4P +/- S1P pretreatment on N-cadherin *in vitro*.

## RESULTS

### HUVEC permeability experiments

**Figure 1:**
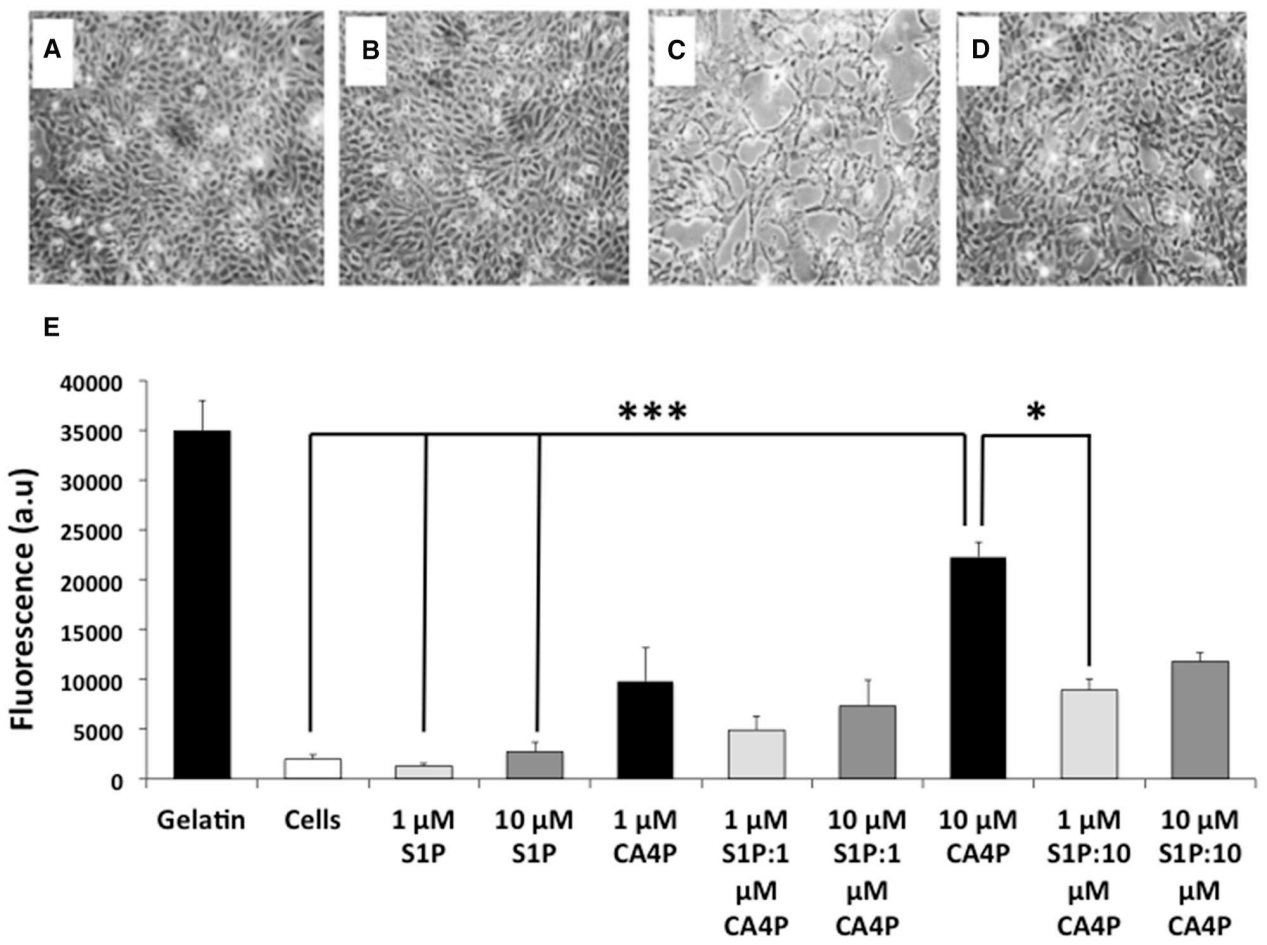
CA4P induced permeability of HUVEC monolayers is ameliorated by pretreatment with S1P **(A-D)**, HUVEC monolayers treated with (A) saline, (B) 10 μM S1P, (C) 10 μM CA4P or (D), 10 μM S1P plus 10 μM CA4P. Gaps in the cell monolayer are clearly visible after treatment with CA4P (C), which are either diminished or not present when cells were pre-treated with S1P (D). **(E)** HUVEC permeability assay using passage of FITC-dextran through the monolayers as a marker of permeability. Cell monolayers and gelatin are negative and positive controls respectively, for FITC dextran egress. Experiments were performed in triplicate three times, and results are means ±SEM. ^***^p<0.005, ^*^p<0.05.

HUVEC grown to confluence on gelatin coated 24 well inserts (3 μm pore size) in 24 well plates remained as intact monolayers after vehicle or S1P treatment (Figure 1A-1B), whereas treatment with CA4P resulted in cell retraction and disruption of the monolayers, with visible gaps formed between the cells (Figure 1C). Cell retraction was attenuated in cultures pre-treated with S1P (Figure 1D).

Untreated cell monolayers, or monolayers treated with 1 μM S1P alone were the least permeable. Treatment with a higher dose (10 μM) of S1P tended to increase the permeability of the monolayer but the increase was not significantly different from controls. Treatment with 10 μM CA4P increased permeability of the monolayers as expected compared to either vehicle alone or S1P treatment (p=<.001). Pre-treatment of cells with 1 μM S1P decreased the 10 μM CA4P-induced permeability (p=0.028) (Figure 1E). These results concurred with the qualitative image data.

### Measurement of red cell flux within fs120 tumours *in vivo*

**Figure 2:**
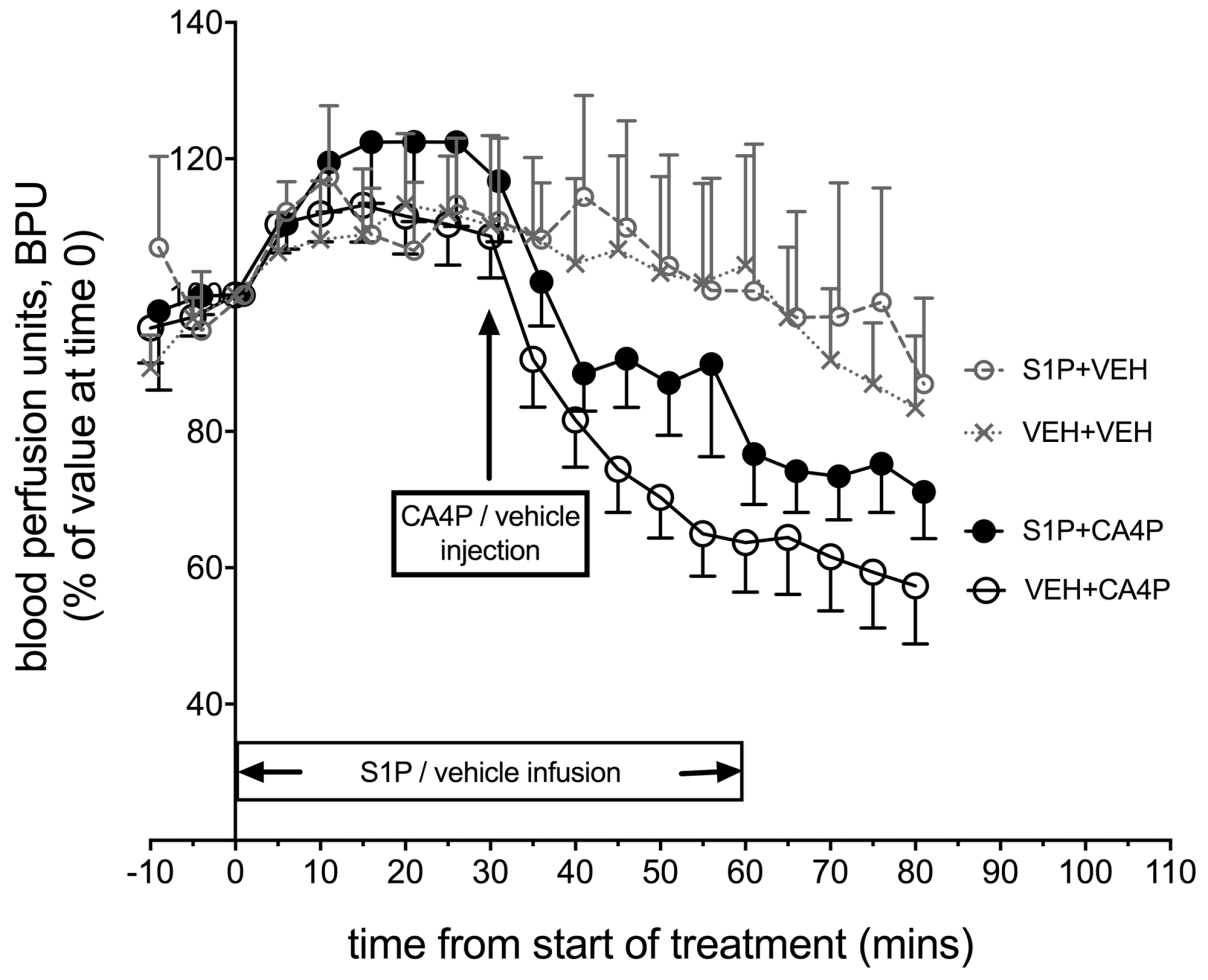
*In vivo* Laser Doppler measurement of blood flow in tumours following treatment with S1P/CA4P Blood perfusion units (BPU) were measured using Laser Doppler flowmetry in fs120 tumour-bearing mice from 4 treatment groups. Results are shown as a percentage of values at time 0, when S1P or S1P vehicle infusion was started (see main text for details). Points represent mean ± SEM for n=9 animals for the vehicle only treated group (VEH+VEH), n=7 for the S1P treated group (S1P+VEH), n=18 for the CA4P treated group (VEH+CA4P) and n=16 for the S1P+CA4P group.

Laser Doppler flowmetry enabled rapid changes in tumour microvascular red cell flux to be measured longitudinally before/after treatment, expressed as blood perfusion units (BPU, Figure 2). Changes were measured in fs120-bearing mice from four treatment groups: S1P vehicle & CA4P vehicle (VEH+VEH; n=9), S1P vehicle & CA4P (VEH+CA4P; n=18), S1P & CA4P (S1P+CA4P; n=16), S1P & CA4P vehicle (S1P+VEH; n=7). Laser Doppler results showed a sudden drop in BPU within minutes after administration of CA4P (30 mg/kg) as expected, which was attenuated, although not significantly, by the prior treatment of animals with S1P. Overall, there was a significant effect of time on laser Doppler signal (P<0.0001) but the effect of treatment did not reach significance (P=0.123). The gradual decrease in BPU (after an initial rise in the first 20-30 minutes) in the vehicle treated and S1P treated groups may relate to anaesthesia-related cardio-respiratory depression over this time period.

**Figure 3:**
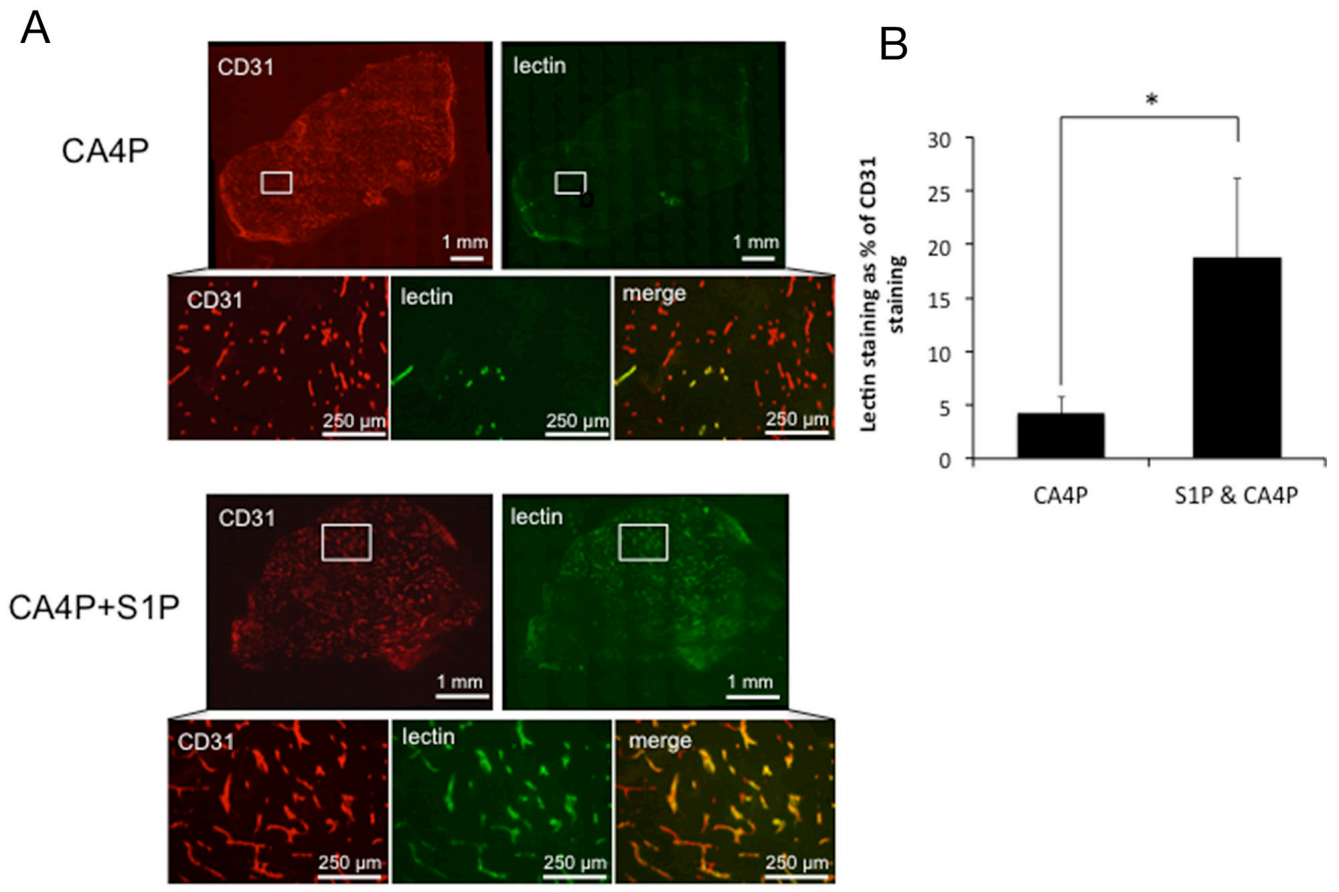
Vascular collapse by measurement of lectin/CD31 staining **(A)** Representative images of staining for CD31 (red) and lectin (green) in tumours treated with CA4P alone or pre-treated with S1P. Merged images reveal fewer perfused vessels in the CA4P treated tumour than in the tumour treated with S1P+CA4P, as indicated by a paucity of lectin staining relative to CD31+ (vascular endothelial) cells **(B)** Perfusion index calculated as the number of pixels stained+ve for FITC-lectin as a % of the number of pixels stained +ve for CD31 for whole data sets in tumours treated with CA4P or S1P+CA4P. Rectangles in the low power images indicate the location of the corresponding high power images. Points and bars represent means ± SEM for n = 7-8 mice. ^*^ p<0.05.

A trend for a protective effect of S1P administration on CA4P-induced vascular disruption using laser Doppler flowmetry encouraged further investigation using CD31/lectin staining of CA4P and S1P & CA4P treated tumours to calculate the tumour perfusion index. CD31^+^ and lectin^+^ areas were measured in stained *ex vivo* tumour sections and lectin^+^ areas expressed as a percentage of CD31^+^ areas. Lectin^+^/CD31^+^ areas were significantly more prevalent in tumours pre-treated with S1P prior to CA4P administration than those treated with CA4P alone (Figure 3), indicating a higher degree of microvascular collapse in CA4P treated tumours without S1P pre-treatment.

### VE-cadherin staining patterns in tumours *ex vivo* following Laser Doppler flowmetry

**Figure 4:**
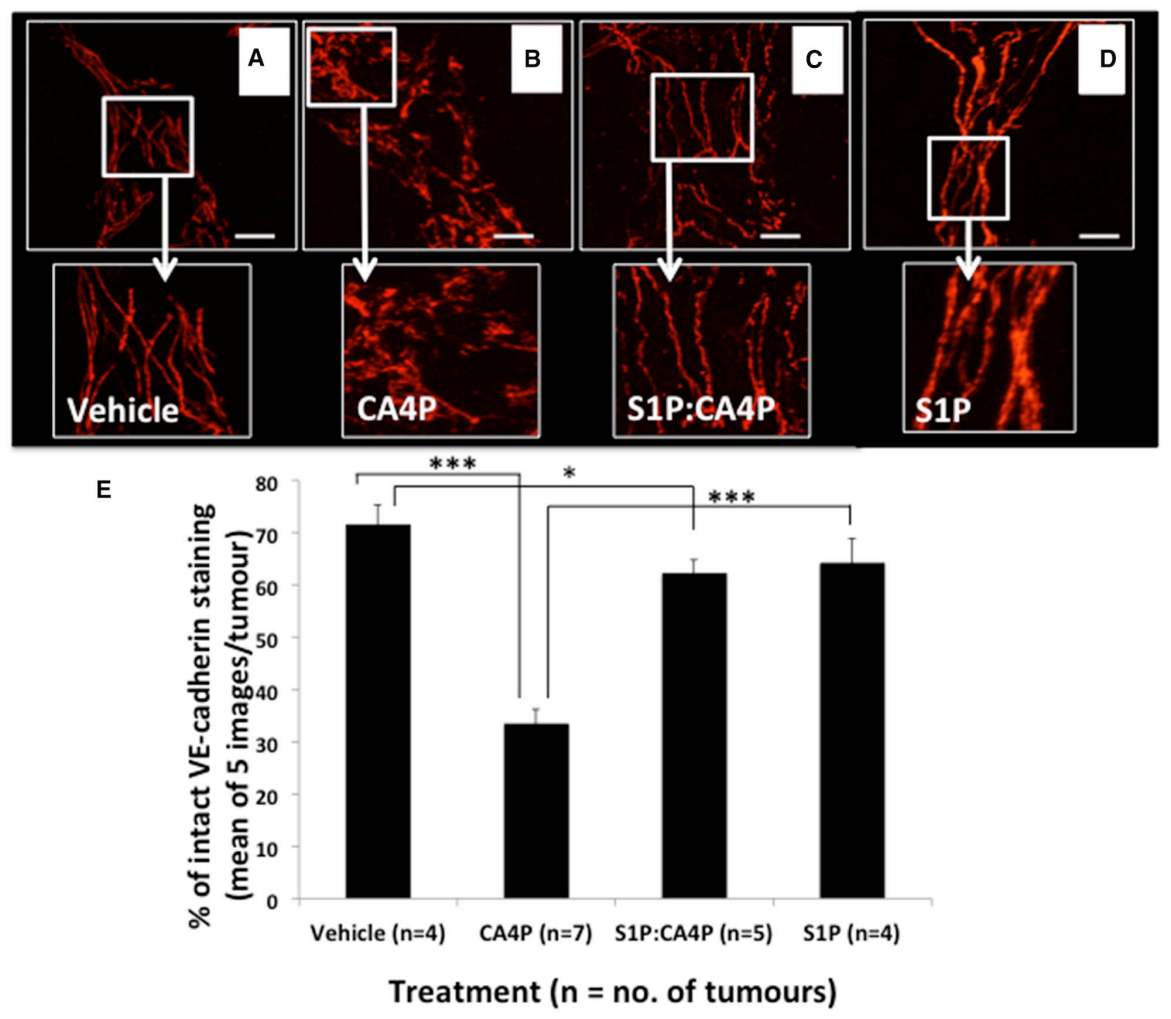
VE-cadherin staining patterns in fs120 tumours excised following S1P/CA4P treatment Tumours were excised from mice treated with vehicles **(A)**, S1P vehicle + CA4P **(B)** S1P + CA4P **(C)** or S1P + CA4P vehicle **(D)**, as described in Methods, and immunofluorescently stained for VE-cadherin. Staining appears smoother and more intact in (A), (C) and (D). Representative images shown from at least five images per tumour; Images show 2D-projections from 3D confocal images viewed using a 40x objective lens. Areas of smooth, intact VE-cadherin were measured as a percentage of total stained area in five images per tumour **(E)**. Data are means ± SEM for the number of tumours shown. ^*^ = p<0.05, ^***^ = p<0.005. Scalebars = 40μm.

Following Laser Doppler flowmetry, tumours were excised from the mice from all four treatment groups, sectioned, and stained for VE-cadherin. Tumours from mice treated with CA4P showed significantly more disruption in VE-cadherin staining than those treated with vehicles, S1P or S1P followed by CA4P (Figure 4A-4D). Image analysis reveals that only ~30% VE-cadherin staining remained intact in CA4P treated tumours versus 65-75% in S1P or vehicle treated. Pretreatment with S1P prior to administration of CA4P increased intact VE-cadherin staining to ~60% of total staining, comparable to vehicle or S1P treated tumours (Figure 4E). It was noted that microvessels found centrally within the tumour mass generally displayed more disrupted VE-cadherin staining after CA4P treatment than those at the tumour periphery (Supplementary Figure 1).

### Flow cytometry of S1P/CA4P treated HUVEC

**Figure 5:**
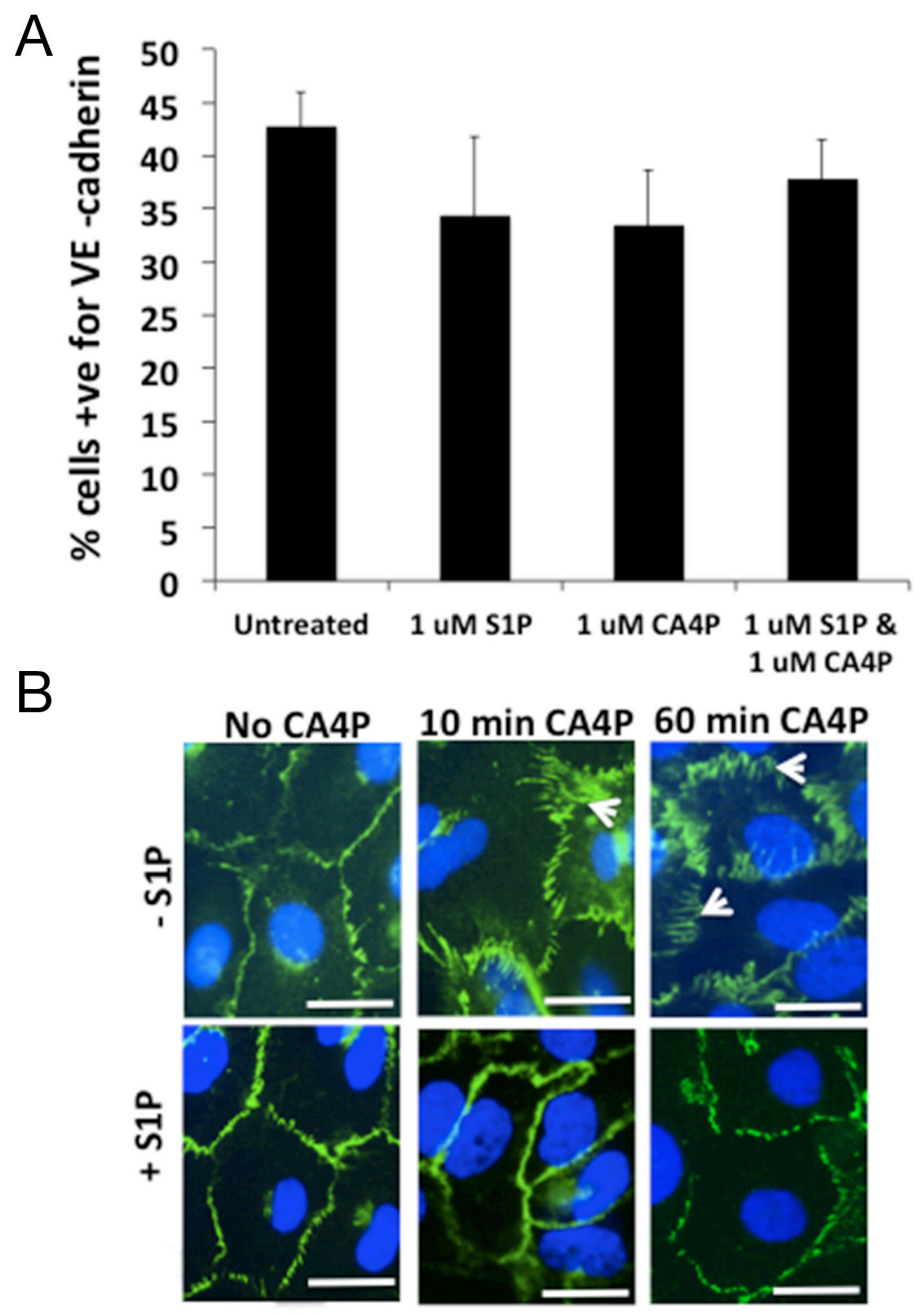
CA4P treatment does not alter the quantity of VE-cadherin expressed on HUVEC cell surfaces FACS analysis of S1P/CA4P treated HUVEC labelled for VE-cadherin reveals that there is no statistical significance in level of expression of extracellular VE-cadherin on treated HUVEC monolayers **(A)**. Bars represent means ± SEM for 3 repeated experiments. Fluorescence immunohistochemistry using an antibody against VE-cadherin on treated HUVEC monolayers shows that extracellular VE-cadherin expression is ‘re-arranged’ rather than downregulated **(B)**. In untreated or S1P-treated cells, VE-cadherin expression is localized to the outer edges of the cells, whereas 10 min after CA4P treatment the VE-cadherin is rearranged into a zigzag pattern (arrowheads), an effect which is decreased by pretreatment with S1P. The change in expression is more prominent after 60 min CA4P treatment but again the effect is somewhat curtailed by S1P pre-treatment. Scalebars = 5 μm.

To determine whether CA4P treatment results in a loss of VE-cadherin expression from the cell surface, adherent HUVEC were treated +/- CA4P, with or without S1P pre-treatment, then detached from the tissue culture plastic and stained in suspension for extracellular VE-cadherin and analysed by FACS. The results showed no difference in total surface expression of VE-cadherin between the treatment groups (Figure 5A) suggesting that the spatial rearrangement of the VE-cadherin following CA4P treatment is responsible for the increased monolayer permeability observed *in vitro* and not a loss of surface localized VE-cadherin. To visualize whether this was indeed the case, HUVEC were grown to confluence on 4-chamber slides and treated with CA4P either with or without S1P pre-treatment and stained for VE-cadherin. In untreated cells, VE-cadherin expression is localized to the outer edges of the cells (Figure 5B, top left), whereas 60 min after CA4P treatment the VE-cadherin redistributed into a zigzag pattern (arrowheads) (Figure 5B, top right), a feature which was not as prominent when cells were pretreated with S1P (Figure 5B, bottom right). This visible redistribution of VE-cadherin following CA4P treatment is consistent with previous results [15]. The change in expression pattern was also evident after only 10 min CA4P treatment (Figure 5B, top middle), which again was curtailed by S1P pre-treatment (Figure 5B, bottom middle). S1P treatment alone had no visible effect on VE-cadherin staining (Figure 5B, bottom left).

### S1P stabilizes microtubules as well as VE-cadherin junctions

**Figure 6:**
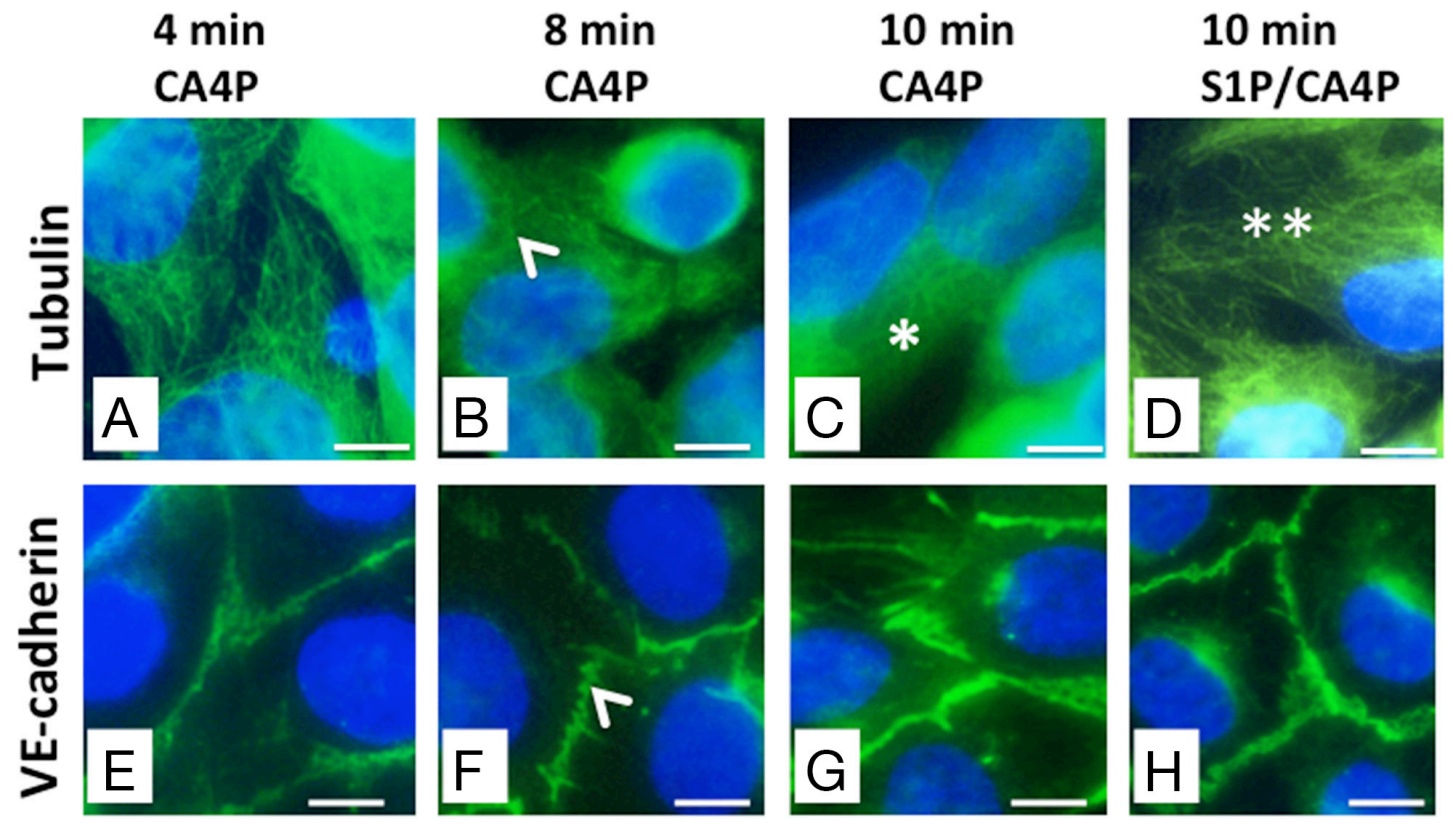
CA4P-induced tubulin depolymerisation as well as VE-cadherin redistribution is prevented by S1P HUVEC monolayers were treated with 1 μM CA4P for 4 **(A, E)**, 8 **(B, F)**, or 10 **(C, G)** minutes then fixed and stained for either tubulin **(A-D)** or VE-cadherin **(E-H)**. HUVEC were also pretreated with 1 μM S1P prior to CA4P treatment; (D,H) show tubulin and VE-cadherin staining following S1P pretreatment followed by 10 minutes CA4P treatment. Tubulin disruption and VE-cadherin rearrangement are both evident from around 8 minutes post CA4P treatment (arrowheads). Tubulin disruption is pronounced by 10 minutes after starting CA4P treatment (^*^) but tubulin is protected at this timepoint by pretreatment with S1P (^**^). Scale bars = 5μm.

The primary action of CA4P on endothelial cells is to depolymerize tubulin, which triggers actin re-organisation into stress fibres, actinomyosin contractility and formation of focal adhesions [12]. HUVEC monolayers were stained for either tubulin or VE-cadherin following different incubation times with 1 μM CA4P (2-10 minutes) to obtain a time-course of tubulin and VE-cadherin effects (Figure 6A-6H). Results showed no effects on either the tubulin cytoskeleton or VE-cadherin junctions for 2 or 4 minute incubation times (4 minute incubation results are shown in Figure 6A and 6E). However, both disruption of microtubules and early spatial rearrangement of VE-cadherin were observed for 8 or more minutes (Figure 6B and 6F; 6C and 6G) and both tubulin and VE-cadherin were stabilized by pretreatment with S1P (Figure 6D and 6H respectively).

### S1P protects against N-cadherin junction disruption by CA4P

**Figure 7:**
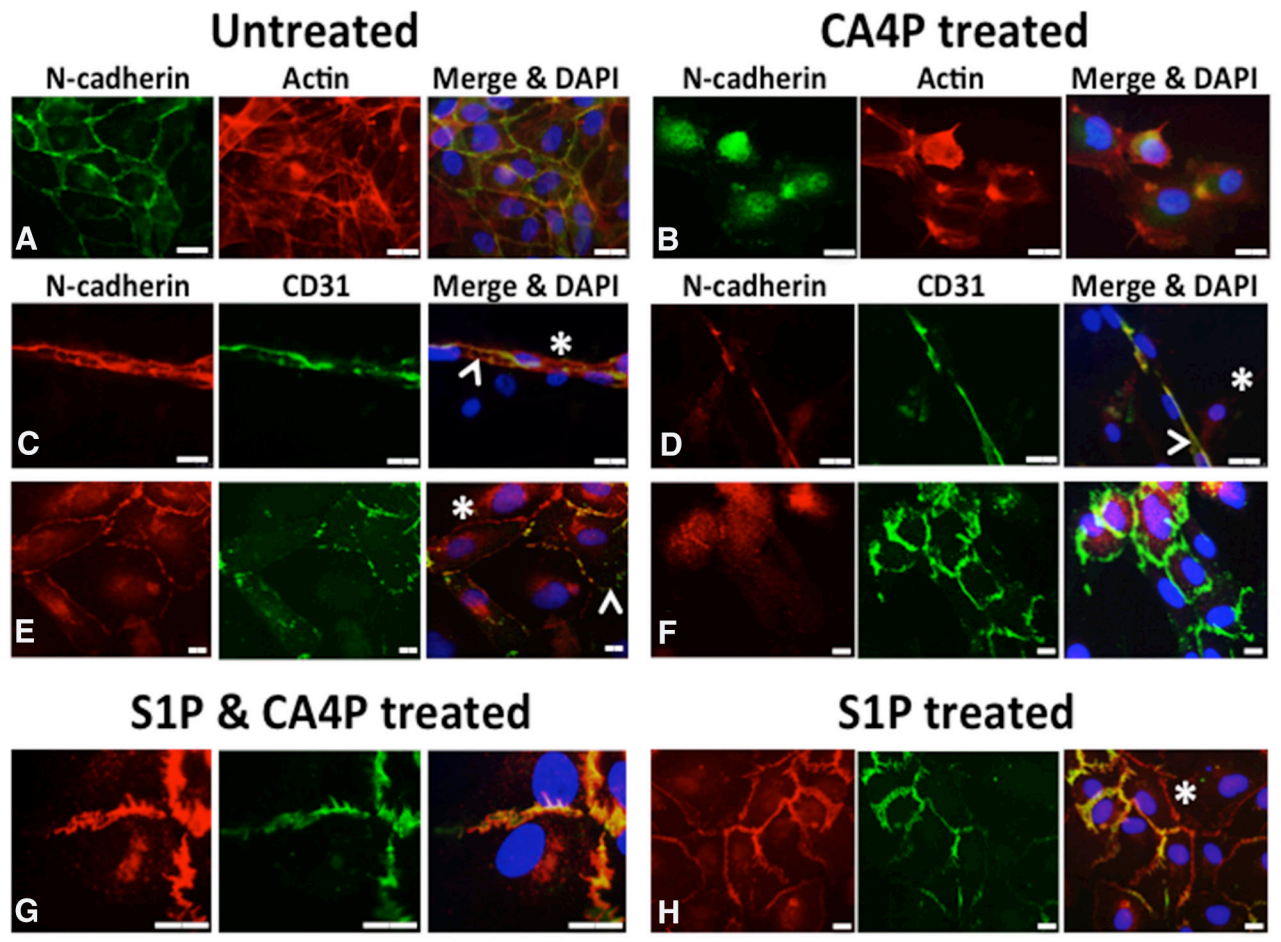
CA4P disrupts N-cadherin adherens junctions Untreated co-cultured HUVEC and vSMC stained for CD31 (indicating endothelial cells) and N-cadherin (indicating EC/SMC junctions) display mostly intact CD31 and N-cadherin staining at intercellular junctions **(A, C, E)**, although some cytoplasmic N-cadherin is also evident. Staining for F-actin in (A) and **(B)**, indicated in red, reveals the cytoskeleton of the cells. (A) shows untreated co-cultures in monolayer stained for N-cadherin (green) and actin (red), whilst (B) shows CA4P treated cells. (C) shows a tubular structure, typically formed from around 40% of the co-cultured cells, with the rest forming flat monolayers as in (E); N-cadherin (red) and CD31 (green). CD31 staining was relatively unaffected by CA4P treatment (green staining in **(D)** and **(F)** versus (C) and (E)). However, N-cadherin staining appears considerably more intracellular and punctate following CA4P treatment (green staining in (B) versus (A) and red staining in (F) versus (E). The change in cellular distribution of N-cadherin following CA4P treatment was blocked by pre-treatment with 1μM S1P, allowing N-cadherin to retain its intercellular location, although its appearance was less smooth than in untreated cells (red staining in **(G)** versus (F)). Cells treated with S1P alone also retained intercellular location of N-cadherin, but with a less smooth appearance than in untreated cells (red staining in **(H)** versus (E)). Yellow in merged images indicates co-localisation at cell-cell junctions. Arrowheads = EC, asterisks = SMC (revealed by lack of CD31 staining). Scalebars = 15μm.

Investiture by mural cells, such as pericytes, is important in the stabilisation of vasculature. The adherens junctional protein N-cadherin is typically located at intercellular junctions between EC and mural cells (pericytes, SMC) as well as EC/EC junctions. We co-cultured HUVEC with human vascular SMC (vSMC) and treated them with CA4P, with or without S1P pre-treatment, then used immunofluorescence to localize N-cadherin. Untreated co-cultured HUVEC and vSMC that were stained for CD31 (indicating endothelial cells) and N-cadherin (indicating EC/SMC junctions) displayed smooth, intact CD31 and N-cadherin staining at intercellular junctions and cell peripheries, with some cytoplasmic N-cadherin apparent (Figure 7A, 7C, 7E). Tubule structures formed from around 40% of the co-cultured cells (the remainder presented as flat monolayers). Treatment for 10 min with 1μM CA4P caused a loss of visible N-cadherin from the cell-cell junctions in the tubule formations (comparing Figure 7C and 7D). CA4P treatment did not appear to affect CD31 distribution, whereas N-cadherin staining appeared intracellular and punctate following CA4P treatment (comparing Figure 7E and 7F, Supplementary Figure 2). The change in N-cadherin localisation following CA4P treatment was blocked by pre-treatment with 1μM S1P, although staining was not so smooth, an effect also observed for treatment with S1P alone (Figure 7G). Supplementary Figure 2 shows N-cadherin staining for endothelial cell only monolayers. Here, there was no peripheral staining, confirming that peripheral staining in the co-cultures was due to endothelial-vSMC interactions.

## DISCUSSION

We hypothesized that stabilization of the microvasculature of fs120 tumours by administering S1P would make them less susceptible to CA4P. *In vitro*, endothelial cell monolayers were less permeable after CA4P treatment when pre-treated with S1P, and VE-cadherin staining of CA4P treated HUVEC demonstrated a surface rearrangement of VE-cadherin, which was prevented by pre-administration of S1P. Following these *in vitro* experiments, we showed a trend towards S1P infusion reducing the *in vivo* drop in laser Doppler flowmetry signal caused by CA4P treatment alone. Convincing evidence for S1P pre-treatment imparting a degree of protection to the tumour vasculature against CA4P-induced shutdown was provided by intravenous administration of fluorescent lectin to calculate a tumour perfusion index. Furthermore, fluorescent immunohistochemical staining of the vasculature from these tumours showed that *in vivo*, pre-treatment with S1P did indeed stabilize VE-cadherin on adherens junctions. Laser Doppler flowmetry is an invasive technique, which cannot readily discriminate between viable and necrotic regions within tumors and only samples a few locations within each tumour. Less invasive techniques that provide relatively high resolution tomographic images of tumor vascular function, such as Doppler ultrasound, dynamic contrast-enhanced magnetic resonance imaging (DCE-MRI) or emerging modalities such as high resolution multispectral optoacoustic tomography (MSOT) [27] would be useful in future studies to investigate the tumor microcirculation in its entirety. Our observation that CA4P has a greater effect upon the smaller microvessels deeper within the tumour mass than the larger vessels found at the tumour periphery is confirmatory of previous data on the action of CA4P [11]. The fact that pre-treatment of mice with S1P could not completely block the CA4P-induced reduction in tumour perfusion could be down to a number of factors. For instance, complete protection of VE- and N-cadherin junctions may not have been achieved. In addition, part of the vascular shut-down instigated by CA4P is likely to be due to other mechanisms such as a direct vasoconstrictive effect on tumour-supplying arterioles [28–29].

The sphingolipid S1P has recently been gaining interest as it plays several roles in both inflammation and cancer-related pathways [20]. Whilst ligation of S1P to its receptor S1P1 at concentrations of 10 nM – 2 μM enhances barrier function by stimulating assembly of adherens junctions [22], Shikata *et al* showed that *in vitro*, binding of high concentrations of S1P (>5 μM) to S1P1 results in active cytoskeletal rearrangement, stress fibre formation and disruption of endothelial barrier integrity [30]. In the current study, 1 μM S1P tended to be more protective than 10 μM S1P against 10 μM CA4P (Figure 1E), consistent with this previous finding.

S1P has been shown to stabilise vasculature via adherens junctions by inducing the translocation of VE-cadherin to endothelial cell junctions [21] and preventing the platelet activating-factor induced rearrangement of VE-cadherin [24]. A recent study investigating barrier function of HUVEC in culture concluded that activation of RhoA was responsible for S1P-induced endothelial barrier enhancement [31]. Interestingly, activated RhoA-Rho kinase is strongly implicated in the damaging effects of CA4P on HUVEC [12] and we recently reported that *in vivo* administration of a Rho kinase inhibitor partially protected tumour blood vessels against CA4P-induced shut-down [13]. It is possible that strong activation of the RhoA-Rho kinase pathway by CA4P has damaging consequences on endothelial cells, whereas activation of this pathway at the endothelial cell periphery by moderate doses of S1P elicits more subtle effects, giving rise to local activation of the actin cytoskeleton and focal adhesions, thereby increasing junctional stability.

Vascular stabilization from investiture of mural cells such as pericytes occurs in the tumour microenvironment as well as ‘normal’ vasculature. S1P stabilizes vasculature in part through its action on N-cadherin, a cell-cell adhesion molecule, which is involved in the adhesion of vessel-stabilizing mural cells to the endothelial cells. Activation of N-cadherin via the G_i_/Rac1 signalling pathway by binding of S1P to S1P1 traffics N cadherin to the cell surface and also appears to increase its adhesive properties [32]. Whilst we have not extensively examined the relative contributions of N-cadherin and mural cells to stabilization of the microvasculature in fs120 tumours in the current study, our data suggest that CA4P treatment disrupts N-cadherin adherens junctions as well as VE-cadherin, and that these junctions can also be protected by pre-treatment with S1P.

Our group has previously shown [26] that fs188 tumors (counterparts to fs120 tumors that express the VEGF188 isoform of VEGF-A in isolation) have a higher degree of investiture of pericytes in the microcirculation, which is likely to be a major factor in fs188 tumours’ relative resistance to CA4P. Stabilisation of N-cadherin junctions in the more sensitive fs120 tumours via S1P, rendering them less susceptible to CA4P, would support this. S1P1 null mice are embryonic lethal, in part due to abnormal mural cell coverage of the dorsal aorta [33], and S1P1 null embryos display atypical N-cadherin localization [34]. It is ligation of S1P to its receptor on EC, not mural cells, that plays a role in regulating pericyte coverage in neovasculature, [34], and *in vitro*, S1P binding to S1P1 on EC is necessary for EC/pericyte interaction [28]. We observed that HUVEC in monoculture alter their distribution of N-cadherin following CA4P treatment from diffuse and cytoplasmic to more nuclear-concentrated, a phenomenon which is blocked by pretreatment with S1P. This supports the hypothesis that CA4P does disrupt N-cadherin as well as VE-cadherin, and that S1P signaling through EC not SMC is important for the regulation of N-cadherin localization.

CA4P is primarily a microtubule depolymerizing agent, and we questioned whether it had direct effects on VE-cadherin or whether the disruption of the VE-cadherin complex is secondary to the action of CA4P on tubulin of the cytoskeleton. We stained HUVEC monolayers for VE-cadherin and tubulin at early timepoints after CA4P treatment, and observed that both tubulin disruption and early VE-cadherin rearrangement were evident by 8 minutes of CA4P treatment. From this data we cannot be certain which is affected first, and a more detailed investigation would be required to elucidate the chain of events, but it was of note to find that pretreatment with S1P appeared to stabilize tubulin, as well as VE-cadherin, against the effects of CA4P. We can only speculate about the pathways involved here. As noted above, signaling through the S1P receptors following ligation of S1P plays a significant role in regulation of the Rho family of GTPases, part of the Ras superfamily of small GTPases [35]. Rac1, a member of the Rho family, is activated by ligation of S1P to S1PR1 in HUVEC [22, 36]. This can lead to phosphorylation of stathmin [37], a process that inhibits stathmin's role in microtubule destabilization [38, 39]. Alternatively, S1P-mediated activation of N-cadherin has been shown to induce microtubule polymerization in EC via a Rac1 pathway [26]. It is conceivable that S1P has vessel-stabilising effects by both activating N-cadherin, thereby strengthening intercellular interactions, and by preventing microtubule destabilization.

Altogether our data suggests that an important component of the VDA CA4P's activity is disruption of VE- and N-cadherin complexes at inter-endothelial cell and EC/mural cell junctions, rendering the vessels prone to haemorrhage and shut-down. Abnormalities of these junctions in tumors could at least partly explain the relative susceptibility of tumour versus normal tissue blood vessels to CA4P. S1P has a protective effect on both adherens junctional proteins as well as the cytoskeletal protein tubulin, and *in vivo*, protects tumour microvasculature against the effects of CA4P. Therefore, antagonism of S1P receptors, in order to diminish the effects of endogenous S1P at the tumour site, may improve the CA4P induced disruption of the microvasculature. Further studies of this pathway are warranted to optimize clinical translation of VDA therapy.

## MATERIALS AND METHODS

### Ethics statement

All experiments were conducted in accordance with the United Kingdom Home Office Animals (Scientific Procedures) Act 1986, local ethical approval and following the UK National Cancer Research Institute's Guidelines for the Welfare and Use of Animals in Cancer Research [40].

### Cell culture

HUVEC (Promocell, Heidelberg, Germany) were cultured on gelatin-coated tissue culture plastics with endothelial cell basal medium (Promocell, Heidelberg, Germany), supplemented with 10% foetal calf serum, 4 μl/ml endothelial cell growth supplement, 0.1 ng/ml human epidermal growth factor, 1 ng/ml human basic fibroblast growth factor, and 1 μg/ml hydrocortisone (all Promocell, Heidelberg, Germany). Media were replaced every two days and cells subcultured just before confluence. HUVEC used in experiments were between passages 3-5. For co-culture of HUVEC and SMC, HUVEC (as above) and Human Vascular Smooth Muscle Cells (Promocell, Heidelberg, Germany) were seeded at a 50:50 ratio into 8-chamber slides, 1 × 10^5^ cells per 400 μl per chamber in media as above. Cultures grew for 3 days.

### HUVEC permeability assay

Methods were as described previously [12]. Cell culture filter inserts with a 3 μm pore size (BD Falcon, Oxford, UK) were placed inside 24 well tissue culture plates and coated with 0.2% gelatin (Sigma-Aldrich, Dorset, UK) in PBS for 30 min. HUVEC were plated inside the filters at a density of 10^5^ cells per insert in 200 μl medium. 700 μl medium was added to each lower chamber and the cells incubated at 37°C, 5% CO_2_ until confluent. Cells were treated with either S1P (Biomol, Exeter, UK) or S1P vehicle (7% methanol in PBS) by directly adding the drugs to the medium for 30 min, at which time CA4P, kindly provided by OxiGene Inc. (now Mateon Therapeutics) or vehicle (PBS) were added. After a further 60 min, media was carefully removed from the inserts to avoid damage to the monolayers and replaced with medium containing 0.8 mg/ml 70kDa FITC-Dextran (Sigma-Aldrich, Dorset, UK). The filters were placed into new wells containing 700 μl fresh medium and plates returned to the incubator for 30 min. The filters were then removed from the wells, contents of the lower wells mixed and fluorescence read using a plate reader (FluoStar) at excitation 485 nm, emission 520 nm. As controls, passage of FITC-Dextran through untreated monolayers and gelatin-coated filters without cells were measured. HUVEC were seeded onto gelatin-coated 12-well tissue culture plates and treated in parallel with S1P/CA4P or vehicle in order to visualise the changes to the monolayers. Experiments were performed in triplicate and repeated three times. Images were acquired using a Nikon Eclipse TS100 microscope and Nikon Coolpix 995 camera.

### *In vivo* treatment with S1P/CA4P

Fs120 cells (1 × 10^6^ cells in 0.05ml) were injected subcutaneously into the dorsal sub-cutis of severe combined immunodeficiency (SCID) mice under isofluorane anaesthesia. Tumours were allowed to develop until they reached ~6mm diameter. In order to measure changes in tumour perfusion during S1P/CA4P treatment, relative changes in volumetric tumour microvascular red cell flux were measured using the Oxyflo™ laser Doppler perfusion system (Oxford Optronix Ltd, Oxford, UK). Group sizes were powered towards detecting a difference between perfusion effects of CA4P alone and CA4P combined with administration of S1P. An intraperitoneal CA4P dose of 30 mg/kg was chosen as this was found previously to cause a significant drop in blood flow in fs120 tumours [41]. Fs120 tumour-bearing mice were weighed and restrained. An intravenous (i.v) cannula was inserted into the tail vein, then the mice were anaesthetised with isoflurane whilst restrained on a heat pad, thermostatically-controlled via a rectal probe, for the remainder of the experiment. Four fibre optic laser Doppler probes were inserted into each tumour, and baseline readings of blood perfusion units (BPU) obtained for ~20 minutes until stable readings were obtained. Throughout the experiment BPU were recorded every 5 minutes. S1P (at 8mg/kg/hr for 20 mins followed by 2mg/kg/hr for 40 minutes) or vehicle (7% methanol in PBS/4% bovine serum albumin for 20 minutes then 1.5% methanol in PBS/4% bovine serum albumin for 40 minutes) was then administered via an i.v line. 30 mins after starting infusion of S1P, a single bolus of CA4P (30mg/kg in heparinised saline) or vehicle (0.3ml of heparinised saline) was injected via an intraperitoneal (i.p) line. After a further 30 minutes the S1P infusion was stopped (1 h total) and the cannula flushed through with saline.

After a further 30 minutes (60 min after injection of CA4P) 0.2 mg FITC-conjugated Lycopersicon esculentum tomato lectin (Vector Laboratories, Peterborough, UK) per mouse was injected 5 min before killing to detect perfused blood vessels at the time of lectin injection. The animals were then killed by an i.v. overdose of sodium pentobarbitone. BPU were recorded for a further 5 minutes to obtain end-baseline readings. For analysis of BPU, results were discarded from any probes which showed inconsistent readings or readings <100. End-baseline readings for each probe were subtracted from probe readings taken throughout the experiment. Data were expressed as a percentage of the pre-treatment value and individual probe readings were averaged at each time point to obtain a single timeline of results for each tumour, which were used for statistical analysis. Means ± SEM of these single timelines were plotted in Figure 2. Immediately following completion of the experiment, the mice were perfused transcardially with 1% paraformaldehyde (PFA) for 2 minutes. The tumours were excised, and half snap frozen for lectin analysis. The other half was placed in 1% PFA for 1 hr then transferred to a sucrose solution for overnight refrigeration prior to processing and embedding for subsequent immunofluorescence studies.

### Immunofluorescence and immunohistochemistry

To stain VE-cadherin or tubulin on HUVEC, HUVEC (Promocell, Heidelberg, Germany) up to passage 5 were grown on gelatin coated 4-chamber slides (Lab-Tek, Nunc, Rochester, New York) to confluence. Cells were fixed in 3.7% formalin in HBSS, washed in PBS, permeabilised in PBS/0.1% Triton-x100 for 5 minutes then washed again in PBS. Slides were then blocked at room temperature for 1h with PBS containing 2% BSA and 5% goat serum, then incubated overnight at 4°C with mouse anti-human CD144 (555661, BD Pharmingen, Oxford, UK) or mouse anti-human tubulin (T4025, Sigma-Aldrich, Dorset UK) at 1:200 in blocking solution. The following day slides were washed in PBS then incubated with biotinylated goat anti-mouse antibody (Vector Laboratories, Peterborough, UK) at 1:100 in blocking buffer for 1h at room temperature. Slides were washed again then incubated with fluorescein avidin (Vector Laboratories, Peterborough, UK) at 1:250 plus Texas Red phalloidin (Invitrogen, Carlsbad, USA) in 10 mM HEPES, pH 7.5, 0.15M NaCl for 1h at room temperature. Finally slides were washed in PBS then mounted in Vectashield with DAPI (Vector Laboratories, Peterborough, UK). Images were acquired using a LeicaDMI4000B inverted microscope and LSAF software. For visualization of VE cadherin in HUVEC cells following S1P or CA4P treatment, cells were pre-treated with either S1P (1 μM) or vehicle (7% MeOH) for 30 minutes, at which point CA4P (1 μM) or PBS was added to the media for the appropriate time. Cells were fixed in 3.7% formalin in HBSS, then stained using an antibody against VE-cadherin as above.

For double staining of N-cadherin and CD31, the general staining protocol as above was followed. S1P/CA4P treated cells were incubated overnight at 4°C with a cocktail of primary antibodies (mouse anti human N-cadherin BD610920 (BD Pharmingen, Oxford, UK) and rabbit anti CD31 (BD Pharmingen, Oxford, UK) at 1:100 per antibody. The following day cells were incubated for 1h with a cocktail of secondary antibodies (Alexafluor 488 goat anti rabbit (Invitrogen, Carlsbad, USA) and biotinylated goat anti mouse (Vector Laboratories, Peterborough, UK)) at 1:200, then, after washing, for a further 1h with Texas Red streptavidin at 1:200 (Vector). Slides were mounted using Vectashield with DAPI (Vector Laboratories, Peterborough, UK).

To calculate the tumour perfusion index, frozen tumours were sectioned to 10μm thick and immunostained for platelet endothelial cell adhesion molecule (CD31), using Alexa-Fluor 555 for visualization, to detect both perfused and unperfused blood vessels. Detailed methods for staining and image capture are described in Williams et al [13]. Individual captured images were analysed using in-house- developed Matlab-based software. Fluorescence channels were manually thresh-holded for each individual tumour. The perfusion index for each image was calculated as the number of pixels positive for FITC (from FITC-lectin) as a percentage of the number of pixels positive for Alexa-Fluor 555.

To visualize VE-cadherin on *ex vivo* tumour sections, 80 μm thick sections were stained. Rat anti-mouse CD144, (BD Pharmingen, BD550548, Oxford, UK) at 1:100 was incubated overnight in Immunomix (0.3% Triton X-100, 0.1% sodium azide, 0.2% bovine serum albumin in PBS) containing 1% normal goat serum. A fluorescent secondary antibody (AlexaFluor goat anti-rat 555, Invitrogen, Carlsbad, USA), was incubated at room temperature for 5 hours. Images were acquired using a Zeiss LSM510 NLO inverted confocal microscope with a x80 objective. Areas of intact VE-cadherin or disruption (5 images per tumour) as judged by areas of smooth staining (intact) or disjointed staining (disruption) were measured using pixel area measurement tools in Photoshop (Adobe). All image acquisition and analysis was performed blind to treatment group.

### Flow cytometry

HUVEC were treated with 1 μM CA4P for 60 min +/- 30 min 1 μM S1P pre-treatment. Following treatment cells were washed in PBS then detached using 2 mM EDTA at 37°C for 15-20 minutes. Cell suspensions were collected, washed twice in PBS then resuspended in ice-cold Immunomix containing mouse anti-human CD144 (BD Pharmingen, BD555661) antibody at a dilution of 1:100. Cells were incubated at 4°C for 1 h, then washed twice and resuspended in ice cold Immuonomix containing Alexafluor 555 (Invitrogen) at 1:200. Cells were incubated at 4°C for 30 mins then washed twice in cold PBS and resuspended in 300 ul cold PBS before analysis on an LSRII FACS machine (BD).

### Statistics

Results from HUVEC monolayer experiments were tested for significant differences using a one-way ANOVA with a post-hoc Tukey test. A 2-way ANOVA, with repeated measures and a post-hoc Tukey test was used for Laser Doppler flowmetry results. Tumour perfusion results were tested with an unpaired Students t-test. All tests were carried out using GraphPad Prism 6 software for Mac OS X (GraphPad Software Inc., San Diego, USA). In all cases, differences between treatment groups were described as significant if the probability corresponding to the relevant statistic was less than 0.05.

## SUPPLEMENTARY MATERIALS FIGURES AND TABLES



## Abbreviations

BPU: (blood perfusion units)
CA4P: (Combretastatin A-4 phosphate)
EC: (endothelial cells)
fs: (fibrosarcoma)
FACS: (fluorescence activated cell sorting)
HBSS: (Hanks balanced salt solution)
HUVEC: (human umbilical vein endothelial cells)
PBS: (phosphate buffered saline)
SMC: (smooth muscle cells)
SCID: (severe combined immunodeficiency)
S1P: (Sphingosine-1-phosphate)
VDA: (vascular disrupting agent)
vSMC: (vascular smooth muscle cells)
VE-cadherin: (vascular endothelial cadherin)
VEGF: (vascular endothelial growth factor)
