# Supplementary material for: The protective role of sphingosine-1-phosphate against the action of the vascular disrupting agent combretastatin A-4 3-O-phosphate
Source: Oncotarget. 2017 Sep 21;8(56):95648–61. doi: 10.18632/oncotarget.21172 (PMC5707050; doi:10.18632/oncotarget.21172)
Supplement: Supplementary file 1 [file oncotarget-08-95648-s001.pdf]

The protective role of sphingosine-1-phosphate against the action of the vascular disrupting agent combretastatin A-4 3-*O*-phosphate

SUPPLEMENTARY MATERIALS

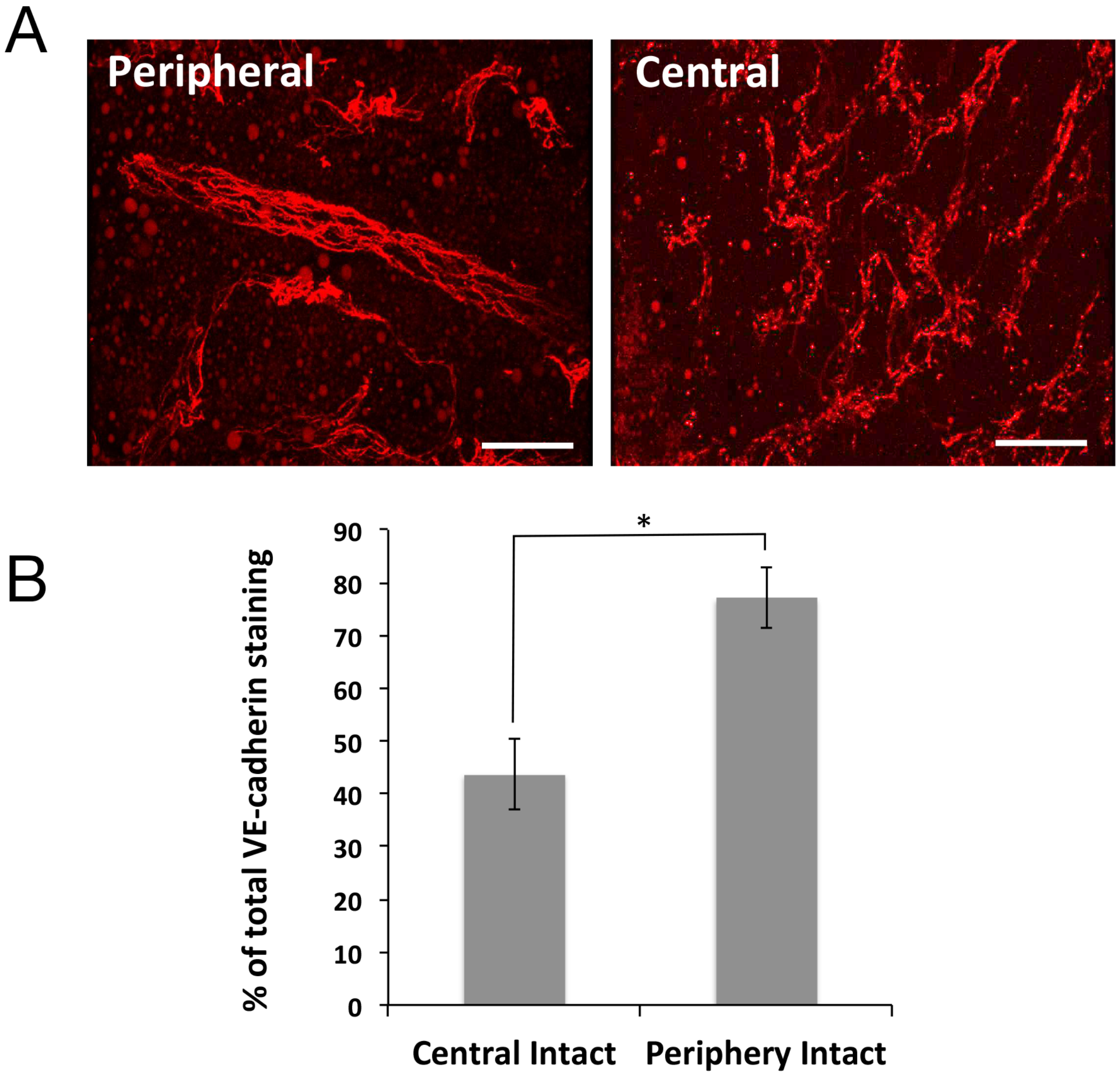

Supplementary Figure 1: VE-cadherin staining of microvessels in central vs peripheral areas of CA4P treated tumours. Microvessels in the central areas of tumours displayed more disrupted VE-cadherin staining following CA4P treatment (30mg/kg) than those found in the tumour peripheries. Frozen sections from 5 tumours were stained for VE cadherin and images acquired on a confocal microscope. **(A)** Representative images of VE-cadherin staining in the peripheral and central tumour regions. **(B)** Three images per tumour were analysed by measuring the area of intact or disrupted VE-cadherin staining and calculating the totals of intact or disrupted staining as a percentage of total VE-cadherin positive area. Scale bars = 40μm, Data shown are means ± SEM, $^{*}p=0.025$ (paired t-test).

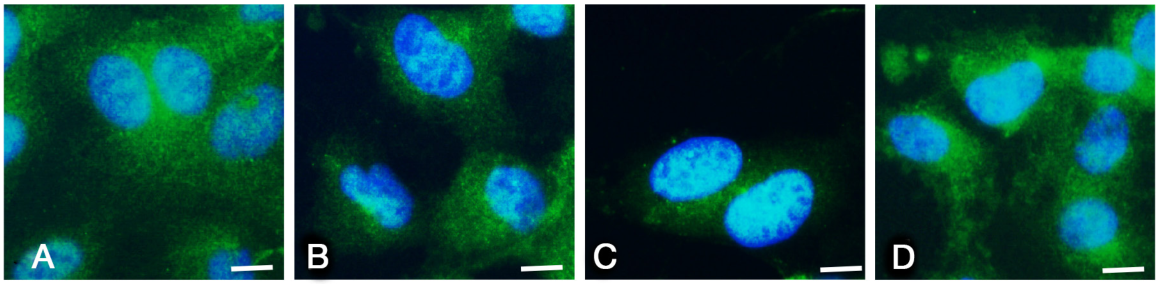

Supplementary Figure 2: HUVEC in monolayer were left untreated **(A)**, treated with 1 μM CA4P for 10 min **(B)** or 60 min **(C)** or pre-treated with 1 μM S1P prior to CA4P treatment **(D)** and stained for N-cadherin (green) and cell nuclei (DAPI, blue). Scalebars = 10 μm.
